# On Catarrhus Vesicæ

**Published:** 1829-07-01

**Authors:** 


### On the Catarrhus Vesicæ of Old People. By M. Civiale.

A very considerable proportion of elderly people, especially in the upper classes of society, and among sedentary, literary, and luxurious characters, labour under, and are ultimately worn out by chronic inflammation of the bladder, with thickening of its coats, diminution of its capacity, and loss of power in its muscular structure to expel the urine. The evil is increased by the aversion which people evince to make known the nature of the complaint before it has made considerable progress—and, in fact, till it has become incurable, as it is generally thought to be by medical practitioners. M. Civiale, from the direction which his studies and practice have taken for several years past, has had numerous opportunities of seeing and treating this distressing malady. He has come to the conclusion, that the primary cause of the disease is atony of the muscular coat of the bladder. This organ, he observes, naturally becomes inert in old age, and slowly expels its contents. This inertia is augmented by sedentary habits, the sitting posture, warm and soft seats—and especially by neglecting the calls of nature, and the inclination to empty the bladder. In consequence of these the bladder becomes distended, and imperfectly contracts—a quantity of urine remains in the organ, and this remora leads ultimately to inflammation of the lining membrane, and, sooner or later, of all the coats of the organ, which become thickened, while the muscular coat loses its contractility. Hence the difficulty and the pain of making water —hence the ropy or muco-purulent urine. In process of time, an irritative fever is established, and the patient is worn out by a painful and lingering complaint. The indications for treatment are more easily laid down than carried into practice. They are as follows :—to lessen the sensibility of the bladder and urethra, if the patient be very irritable — to facilitate the discharge of the urine—to prevent the accumulation and remora of glairy mucus in the bladder—to alter the disposition of the mucous membrane of that organ, and to draw irritation to the surface. These indications, M. Civiale asserts, are easily accomplished, if we go the right way to work. The catheter, he considers, as the grand instrument in the cure of the disease. By this we not only empty the bladder by removing the mucus and urine, but we can inject diluents that wash away all irritating matters out of the bladder. By these means the contractility of the organ is restored, and the urine is rendered limpid. We shall notice one of the cases brought forward by the author of this paper.

*Case.* An old gentleman of 70 years, wrote to M. Civiale, from Germany, respecting his malady. It had commenced more than a year previously, with the usual symptoms of catarrhus vesicæ, for which he had been under some of the most eminent medical practitioners of the Continent, without benefit. The urine became more and more ropy, fetid, black, and puriform—the pains were constant — the appetite failed—the flesh wasted, and the patient was hardly able to leave his room. Constipation became very troublesome, in consequence of the quantities of opium he was obliged to take. With difficulty he came to Paris, and M. Civiale, while examining him in order to ascertain if there was a calculus, perceived that the bladder never entirely emptied itself—that its internal surface was exceedingly irritable, and that contact with the sound produced great pain. In short, there was catarrhus vesicæ of a very intense kind established. The patient was put on the most unirritating regimen—the opium was discontinued, and lavements employed. The catheter was daily introduced, so as to completely empty the bladder, and inject tepid water in its stead. In 25 days of this treatment, the contractility of the bladder was restored—the urine became limpid —the pains ceased. The patient recovered his appetite and sleep, and departed from Paris cured.

It was in the course of the various operations for breaking down the stone in the bladder, that M. Civiale became acquainted with the beneficial effects of catheterism in catarrhus vesicæ—a common accompaniment of calculus. But it is to be remarked, that this benefit was perceived long before the stone was destroyed or extracted, and, consequently the extraction was not the cause of the relief; but the operation of drawing off the urine, together with the injection of tepid water into the bladder. We are of opinion, that the suggestions of M. Civiale deserve particular attention; since the disease is equally dangerous and intractable.

